# Perspectives of people with spinal cord injury on a pain education resource

**DOI:** 10.3389/fpubh.2024.1385831

**Published:** 2024-06-19

**Authors:** Gabriel E. Fernandez, Kim D. Anderson, Roberta Vastano, Scott I. Frank, Linda E. Robayo, Nicholas P. Cherup, William Kochen, Eva Widerström-Noga

**Affiliations:** ^1^The Miami Project to Cure Paralysis, University of Miami, Miami, FL, United States; ^2^Psychology Graduate Program, Nova Southeastern University, Davie, FL, United States; ^3^Department of Physical Medicine and Rehabilitation, MetroHealth System, Case Western Reserve University School of Medicine, Cleveland, OH, United States; ^4^Neuroscience Graduate Program, University of Miami, Miami, FL, United States; ^5^Department of Neurological Surgery, University of Miami, Miami, FL, United States

**Keywords:** spinal cord injury, chronic pain, pain education, neuropathic pain, survey

## Abstract

**Introduction:**

Spinal cord injury (SCI) often leads to neuropathic pain that negatively affects quality of life. Several qualitative research studies in individuals with SCI who experience neuropathic pain indicate the lack of adequate information about pain. We previously developed an educational resource, the *SeePain,* based on scientific literature and a series of qualitative interviews of people with SCI, their significant others/family members, and SCI healthcare providers.

**Methods:**

However, to quantitatively evaluate the utility of this educational resource in a larger sample, we examined the agreement and usefulness ratings of statements regarding clarity/comprehensibility, content, and format of the *SeePain,* derived from the thematic analysis of our previous qualitative interviews. Participants completed a survey that provided a digital version of the *SeePain* and then rated their agreement/usefulness with the statements using numerical rating scales.

**Results:**

There were overall high perceived agreement and usefulness ratings regarding the *SeePain*’s clarity, content, and format. A factor analysis reduced the agreement and usefulness ratings into 4 components (content, clarity, format, and delivery medium). Group comparisons showed that individuals with higher education were more likely to endorse electronic and website formats, and the usefulness of a shorter version of the SeePain; females and younger individuals showed greater endorsement for clarity. Finally, higher pain intensity ratings were associated with greater agreement and usefulness of the content of the *SeePain*.

**Discussion:**

Overall, these results support the utility of the *SeePain* as a source of information regarding pain that may facilitate communication about pain and its management following SCI.

## 1 Introduction

Individuals with spinal cord injury (SCI) commonly have multiple health complications and it is estimated that between 60 and 70% of them experience neuropathic pain (1). These pain conditions are often difficult to manage and can significantly impact an individual’s quality of life (QoL) (2). Many people with SCI who experience persistent pain report that they do not receive adequate information about their pain and how to manage it (3, 4). A recent qualitative study, conducted by our laboratory, showed that facilitators to better pain management included education regarding neuropathic pain and the various treatment options, and improved patient access to non-pharmacological treatment options (5). Therefore, having a better understanding of pain and available treatment approaches including self-management may help to reduce pain’s negative impact on daily life.

Several pain education programs have been developed to address the informational needs of individuals with SCI and pain, with studies indicating variable results (6–8). Participation in a multidimensional pain management program led to reduced levels of depression and anxiety, while also improving measures of coping and sleep quality (6). Similarly, a cognitive behavioral pain educational program significantly reduced pain intensity and pain-related disability scores, as well as anxiety and increased life participation. However, pain-related measures failed to retain statistical significance in a three-month follow-up assessment (8). It was also shown that individuals who completed an interdisciplinary pain program did not experience a decrease in pain severity, but they reported significant reductions in daily pain interference and a greater sense of life control. Overall, this program positively impacted the participants’ ability to cope with pain and improve overall well-being (7). These results support the value of providing educational programs for managing SCI-associated neuropathic pain, however, more research is needed to determine the most useful format and content.

Recently, we developed an educational resource entitled the *SeePain*, to address the specific informational needs of individuals with SCI and neuropathic pain (9). This resource was developed based on qualitative interviews with people living with SCI and neuropathic pain, their significant others/family members, and healthcare providers specializing in SCI. The *SeePain* offers detailed insights on post-SCI pain and is divided into two separate modules. The first module contains information about categories of pain, underlying mechanisms of pain, the long-term nature of pain, and the psychological and social effects of pain. The second module discusses managing pain, including self-help strategies, and pharmacological and non-pharmacological-based treatments. Both modules incorporate multiple quotes from relevant stakeholders to provide real-life context. The ultimate goal of the *SeePain* is to facilitate communication between patients, their significant others, and healthcare providers, empowering patients with the knowledge and self-management skills to better navigate their pain-related issues (5).

Because the *SeePain* was developed based on a relatively limited sample (9), the present study was designed to quantitatively assess its perceived comprehensibility/clarity, content, format, and overall usefulness in a larger group of people with SCI. To accomplish this, we created an online survey in which we asked participants with SCI who had experienced SCI-related pain for 6 months or longer, to review the *SeePain* in its entirety and respond to statements related to the agreement and usefulness of this resource. Demographic information and pain characteristics were also collected. Our primary aim of the present study was to quantify the agreement and usefulness with the *SeePain* to determine overall utility, underlying dimensions, and any associations with pain or demographic characteristics.

## 2 Methods

### 2.1 Study participants

**Figure 1:**
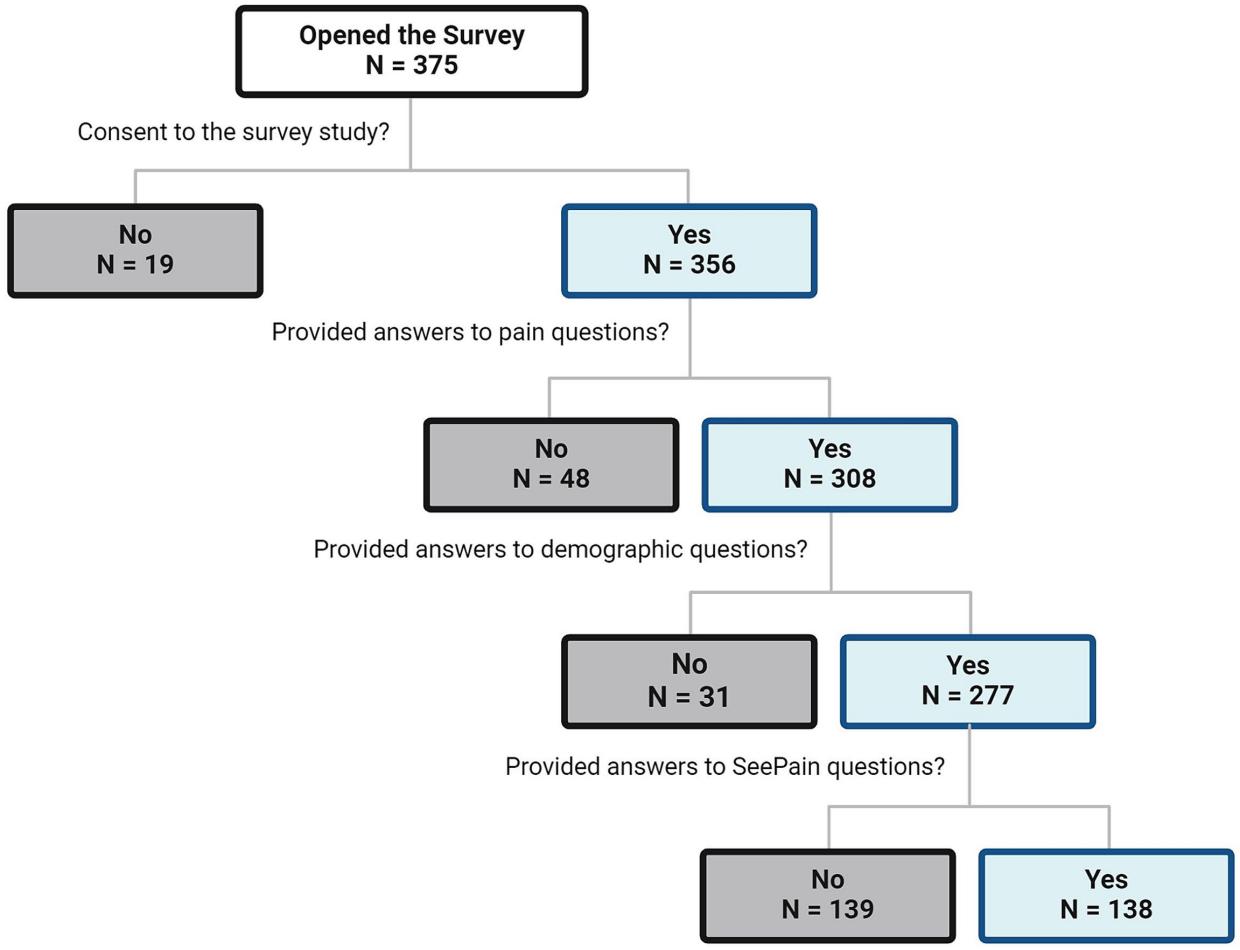
Flowchart of participants’ responses collected.

Participants were invited to complete a survey via email using an existing opt-in listserv of the Miami Project to Cure Paralysis and SCI community networks. Three-hundred and fifty-six men and women aged 18–70, with moderate to severe SCI-related pain [rated as ≥4 on a numerical rating scale (NRS) from 0 (no pain) to 10 (worse pain imaginable)] for 6 months or longer, consented to do the survey. Of these participants, 308 answered the pain-related questions, 277 answered the demographic-related questions, and 138 reviewed the *SeePain* and answered all the survey questions (see Figure 1). There were 139 participants who did not proceed to read the entire document but who provided demographic and pain information (non-completers) and 138 participants who proceeded to read the *SeePain* and complete the entire survey (completers). These two groups were compared on demographic and pain information to determine if the completers were representative of the entire sample (see Tables 1, 2). The study protocol followed ethical guidelines outlined in the Declaration of Helsinki and received approval from the institutional review board at the University of Miami Miller School of Medicine.

**Table 1:** Demographic characteristics of non-completers (*N* = 139) and completers (*N* = 138).

	Non-completers *N* (%)	Completers *N* (%)	p*
**Age range**			0.56
Young (18–45 years old)	50 (36.0)	45 (32.6)	
Older (46+ years old)	89 (64.0)	93 (67.4)	
**Gender**^**a**^			0.17
Male	97 (69.8)	85 (61.6)	
Female	41 (29.5)	51 (37.0)	
Non-binary	0 (0)	1 (0.7)	
Transgender	1 (0.7)	1 (0.7)	
**Ethnicity**^**b**^			0.57
White/Caucasian	106 (76.3)	114 (82.6)	
Hispanic or Latino	12 (8.6)	12 (8.7)	
Black or African-American	11 (7.9)	7 (5.1)	
American Indian or Alaskan Native	5 (3.6)	3 (2.2)	
Asian	3 (2.2)	2 (1.4)	
Native Hawaiian or Other Pacific Islander	1 (0.7)	0 (0.0)	
Multiple Ethnicities	1 (0.7)	0 (0.0)	
**Level of injury**			0.77
Cervical	53 (38.1)	55 (39.9)	
Below cervical	86 (61.9)	83 (60.1)	
**Education**			0.24
Did not finish college or prior	47 (33.8)	56 (40.3)	
College or higher education	92 (66.2)	82 (59.4)	
**Years with SCI**			0.30
5 years or less	18 (12.9)	17 (12.3)	
6–15 years	69 (49.6)	57 (41.3)	
16 years or more	52 (37.4)	64 (46.4)	
**Years with pain**			0.55
5 years or less	23 (16.5)	29 (21.0)	
6–15 years	66 (47.5)	58 (42.0)	
16 years or more	50 (36.0)	51 (37.0)	
**Neuropathic pain**^**c**^			0.70
Yes	104 (74.8)	106 (76.8)	
No	35 (25.2)	32 (23.2)	

^*^Chi-square Test. ^a^Only males and females were compared. ^b^Comparison was performed on White/Caucasian, Hispanic or Latino, Black or African American. ^c^The presence of neuropathic pain was determined based on a total score of 2 or higher in the Spinal Cord Injury Pain Instrument (SCIPI) questions (14).

**Table 2:** Pain characteristics of non-completers (*N* = 139) and completers (*N* = 138).

	Non-completers Mean (SD)	Completers Mean (SD)	p*
International SCI pain basic data set	0–10 scale		
Average pain intensity^a^	6.60 (1.78)	6.44 (1.80)	0.46
Hard to deal with the pain	5.85 (2.26)	5.35 (2.45)	0.08
Total pain interference^b^	6.08 (2.23)	5.73 (2.50)	0.22
Multidimensional pain inventory	0–6 scale		
Pain severity^b^	4.19 (1.18)	3.96 (1.18)	0.11

^a^Of all your pain problems in the past week, ^b^In the last week. *Unpaired *t*-test with Holm-Šídák correction for multiple comparisons.

### 2.2 Survey design

**Figure 2:**
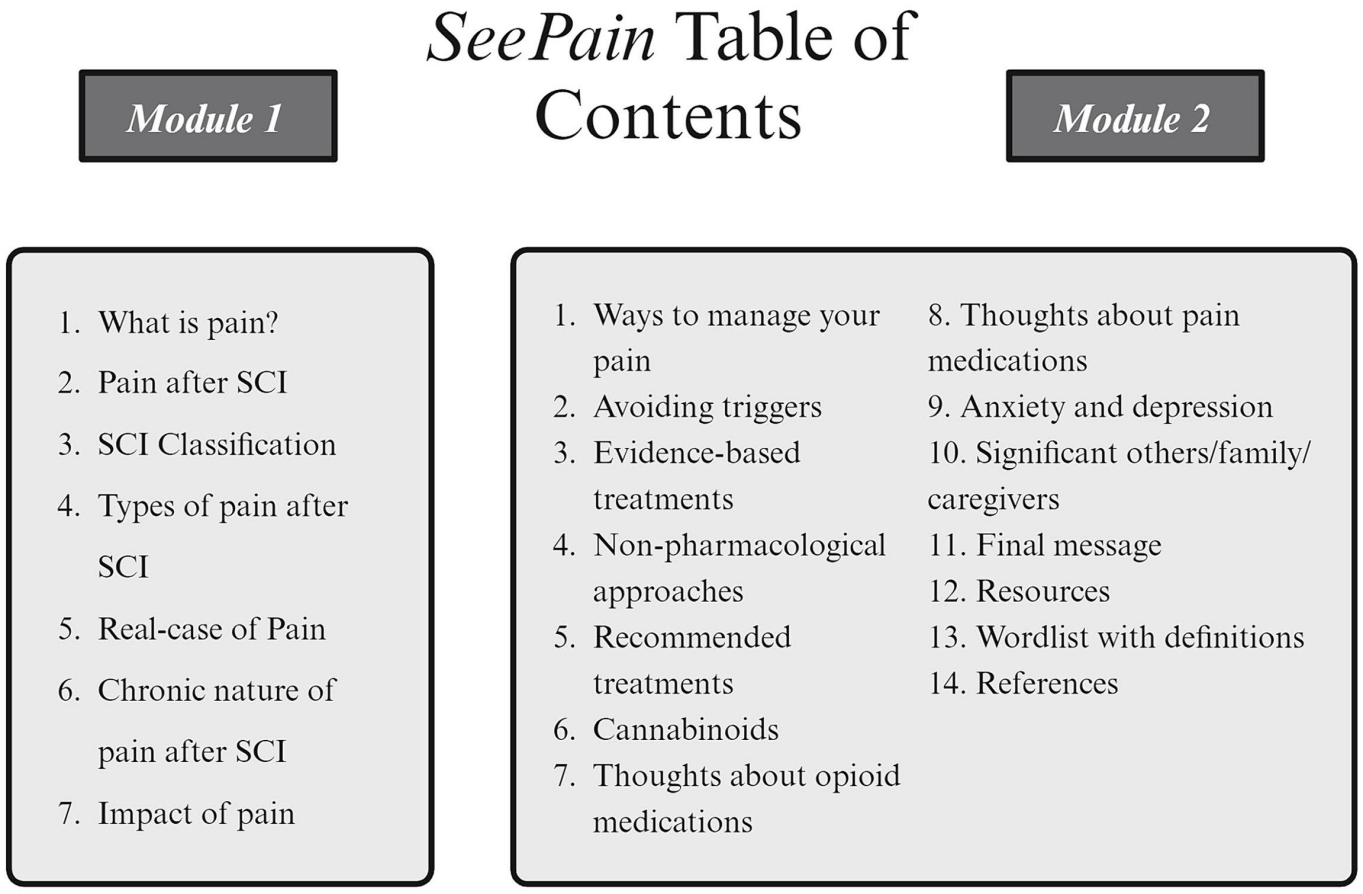
Image of the table of contents from the *SeePain* modules 1 and 2.

The survey was created and administered using the cloud-based software SurveyMonkey™ (San Mateo, CA) (10). For a detailed description of the security features of SurveyMonkey.^1^ The first part of the survey consisted of an information page and an institutionally approved waiver of signed informed consent. Consent was granted by the agreement to continue with the survey. Forty-three individual questions covered demographic factors, pain characteristics, and statements that participants rated on a numerical rating scale regarding their perceived agreement and usefulness with the *SeePain* (see Supplementary material for the full survey). These statements were based on the thematic analysis of qualitative interviews in a previous study Figure 2 (9).

#### 2.2.1 Demographic and injury-related questions

There were eight questions about demographic and injury characteristics. These questions included age range, gender, ethnicity, education, level of injury, sensation or voluntary movement below the level of injury, years living with SCI, and years living with pain.

#### 2.2.2 Pain-related questions

Pain-related questions were obtained from the International Spinal Cord Injury Pain Basic Dataset- version 2 (ISCPBD-2) (11) to evaluate average pain intensity and pain interference with day-to-day activities, mood, and sleep over the last week. These questions were rated on a NRS ranging from 0 (no pain/interference) to 10 (extreme pain/interference). The Multidimensional Pain Inventory Pain Severity subscale (12) was used to evaluate pain severity. This subscale includes three questions answered on a six-point Likert scale with 0 (No Pain/Suffering or not at all severe) to 6 (Extreme Pain/Suffering or extremely severe) and has been validated in the SCI chronic pain population (13). We also assessed difficulty in dealing with pain. This single question provides a rating of how difficult it is to deal with pain on an NRS from 0 (not hard at all) to 10 (extremely hard). Finally, the Spinal Cord Injury Pain Instrument (SCIPI) (10) was used to assess the presence of neuropathic pain. The SCIPI is a concise assessment tool consisting of four items designed to examine the neuropathic pain characteristics among individuals with SCI. Each item requires a “Yes” or “No” response (Yes = 1, No = 0). The total score on the SCIPI ranges from 0 to 4, with a score of 2 or higher suggesting the presence of neuropathic pain (14).

#### 2.2.3 *SeePain* questions

Evaluative statements regarding the *SeePain* were based on the most frequently endorsed themes from qualitative interviews (9). For example, “*I believe that the SeePain should include specific information regarding the risks for opioid addiction*” (4). Based on prior experience and the frequency of themes, 23 evaluative statements and questions were developed for the survey. We used a 10-point Likert scale to investigate the perceived agreement and usefulness ratings of the *SeePain*. Statements and questions about the *SeePain* items were rated from 0 (completely disagree/ least useful) to 10 (completely agree/most useful). A link to the *SeePain* is available at (9).

#### 2.2.4 Participants’ comments

Participants were able to provide comments with respect to the overall comprehensibility/clarity, content, and usefulness of the *SeePain*. These comments were collected to supplement the quantitative agreement and usefulness ratings concerning the *SeePain*.

### 2.3 Statistical analysis

#### 2.3.1 Demographic, injury and pain characteristics

Frequencies and percentages were calculated to represent categories across demographic variables for the non-completers (*N* = 139) and completers (*N* = 138). In addition, we classified participants as having neuropathic pain and non-neuropathic pain. Chi-square tests were used to compare the non-completers and completers across demographic and injury characteristics. For gender, only males and females were compared due to small sample sizes in the non-binary and transgender categories. For ethnicity, we included only White/Caucasian, Hispanic or Latino, Black or African American due to small sample sizes in 4 categories (American Indian or Alaskan Native, Asian, Native Hawaiian or Other Pacific Islander, Multiple Ethnicities).

Means and standard deviations were calculated to indicate pain characteristics among participants in the non-completers and completers groups. Subsequently, unpaired t-tests with Holm-Šídák correction for multiple comparisons were used to compare the non-completers and completers groups across pain characteristics.

#### 2.3.2 Perceived agreement and usefulness of the *SeePain*

Means and standard deviations were calculated to represent overall agreement with each of the six statements regarding the *SeePain*, as well as the overall usefulness of specific areas and formats of this resource. The participants in the completers group (*N* = 138) provided answers to all the questions regarding the perceived agreement and usefulness of the *SeePain*.

#### 2.3.3 Factor analyses of perceived agreement and usefulness ratings

To comprehensively explore the relationships among the perceived agreement and usefulness scores based on their shared underlying dimensions, an exploratory factor analysis was conducted. This analysis encompassed all thematic items, regardless of their average perceived agreement and usefulness ratings, to ensure the inclusion of all relevant factors and an accurate extraction of all potential variance among the variables. To enhance the interpretability of the results, items with cross-loadings that differed by ≤0.15 were eliminated. The adequacy of the factor analyses was assessed using Bartlett’s test of sphericity, which demonstrated a significant chi-square value and a probability of <0.05, indicating a satisfactory fit. The Kaiser-Meyer-Olkin (KMO) measure for sampling adequacy exceeded the threshold of 0.60, further confirming the appropriateness of the factor analyses.

#### 2.3.4 Group comparisons on factors’ scores

Using independent samples *t*-tests we tested whether educational background (graduated from college vs. non-graduated), age (young: 18–45 and older: 46+ years old), and gender (male vs. female - excluding two participants identified as transgender and non-binary as they could not be classified as male or female) were different on factors’ scores of the components derived from the factor analysis. Then, level of injury (cervical vs. below-cervical) and presence of neuropathic pain (neuropathic pain: SCIPI score of 0–1 vs. neuropathic pain: SCIPI score of 2–4) were compared only on those components related to the information, that is clarity/comprehensibility and content, of the SeePain.

Additionally, using one-way ANOVAs,we tested whether years of living with pain [short (5 years or less), medium (6–15 years), and long (16 years or more)], years with SCI [short (5 years or less), medium (6–15 years), and long (16 years or more)], and ethnicity (White/Caucasian, Hispanic or Latino, Black) were different on factor scores of the components derived from the factor analysis; also only on those components related to the information, that is clarity/comprehensibility and content, of the *SeePain*.

#### 2.3.5 Correlations

Finally, we conducted Pearson correlations between the factor scores of the components derived from the factor analysis (clarity/comprehensibility and content, of the *SeePain*) and pain intensity (average pain intensity of all pain problems in the past week), the pain severity subscale, pain interference and difficulty dealing with pain.

All statistical analyses were performed using SPSS 27 for Windows, with a significance level of <0.05 considered statistically significant.

## 3 Results

### 3.1 Demographic and injury characteristics

Demographic and injury characteristics were compared between non-completers and completers (Table 1). Overall, there were no significant differences between those who completed all questions on the survey versus those who did not, this was true for all demographic and injury characteristics that we compared. Specifically, the age span for the sample ranged from 18 to 46 and older, with the largest portion of the sample falling between 46 and older years of age. The gender distribution also revealed approximately equal numbers of males to females across both groups, plus two participants who identified as transgender and non-binary, respectively. In terms of ethnicity, the majority of all participants identified as White/Caucasian. There was also a higher portion of participants with below-cervical injuries compared to cervical injuries. Similarly, there was a higher proportion of participants with college or higher education. Most individuals in both groups also reported living with their injuries for 6 years or longer, indicating that our sample falls under the advanced chronicity stage of their injury. Additionally, years with pain ranged from “5 years or less” to “16 years or more,” with the largest segment for both groups experiencing pain for “6–15 years.” Based on the total score on the SCIPI questions, 75% of non-completers and 77% of completers were classified as having neuropathic pain.

### 3.2 Pain characteristics

Table 2 shows the pain characteristics of the participants. Pain measures were not significantly different between completers and non-completers.

### 3.3 Perceived agreement with the *SeePain*

Table 3 presents an overview of the participants’ level of agreement regarding the *SeePain*. Participants reported high levels of agreement with the overall comprehensibility and clarity of the document with the language being reported as highly understandable, including figures and tables. Information about pain and pain types was rated as being clear and well-explained. Additionally, participants perceived the overall content as useful and relevant to their needs and considered the format of the *SeePain* to be good.

**Table 3:** Perceived agreement with the *SeePain* (*N* = 138).

Statements	Mean (SD)^*^ 0 to 10
The language is understandable throughout the *SeePain*	8.29 (1.97)
Figures and tables are clear	8.28 (2.00)
The overall content is useful and relevant	7.97 (2.29)
The explanations regarding pain and how it happens are clear	7.96 (1.99)
The format of the *SeePain* is good	7.86 (2.21)
The pain types that are common and how they develop over time after SCI are well explained	7.78 (2.06)

^*^0 to 10 scale, 0 = completely disagree and 10 = completely agree.

### 3.4 Perceived usefulness of the *SeePain*

The data presented in Table 4 illustrates the perceived usefulness of the *SeePain*. Participants reported the highest average level of usefulness for the modules regarding SCI-related pain, self-management of pain, communication with healthcare providers, and factors that may make pain worse. In contrast, modules regarding healthcare providers’ perspectives and support for significant others/family received lower usefulness ratings, although these modules were still perceived as moderately useful. Additionally, participants indicated that a shorter version of the *SeePain* could be useful and rated the electronic and website versions of the SeePain to be most useful over a paper version.

**Table 4:** Perceived usefulness of the *SeePain* (*N* = 138).

Statements	Mean (SD)^*^ 0 to 10
**Please indicate areas that were most useful and least useful to you**	
SCI-related pain	7.73 (2.02)
Self-management of pain	7.33 (2.41)
How useful would the *SeePain* be to help you to discuss your pain with your doctor and other healthcare professionals?	7.12 (2.83)
Factors that make pain worse	7.10 (2.35)
Role of psychological factors	6.70 (2.62)
How useful would it be to have a shorter version of the *SeePain*?	6.59 (2.78)
How useful would the *SeePain* be to help you discuss your pain with your significant other and family members?	6.56 (2.81)
Cannabis/opioids	6.50 (2.81)
Pain medication	6.49 (2.70)
Support for significant other/family	6.16 (2.78)
Healthcare provider perspectives	5.97 (2.44)
**Please indicate the formats that are most useful and least useful to you**	
Electronic (current version)	7.78 (2.45)
Website version	7.67 (2.37)
Short videos	6.91 (3.09)
In-person learning	6.07 (3.22)
App-based learning	5.75 (3.30)
Paper version	5.04 (3.56)

^*^0 to 10 scale, 0 = completely disagree and 10 = completely agree.

### 3.5 Participants’ comments

#### 3.5.1 Pain and SCI-related comments

Participants provided comments with respect to their pain and overall experience with SCI. See below for selected quotes:

“It’s important to remember that everyone is an individual and their SCI is unique. Some people cope with pain better than others. It does not make it any easier for them to live with it. And coping with pain over years with no relief or end to look forward to is mentally exhausting. It wears the most positive of us down. I think there is a lot that could be done to help people living with SCI if they were understood and not overlooked.”

#### 3.5.2 *SeePain*-related comments

Participants provided comments with respect to the *SeePain*. Overall, their comments emphasized the utility of the educational material. Participants stated that the information was accurate and well explained, they were also grateful for the material as it provided explanations for those with limited resources and support. See below for representative comments:

“This information puts into words what I try to explain to people. No one understands, even Dr’s, Physical Therapist, etc. Thank you for taking the time and listening to enough of us to make this presentation. Very helpful! And sadly, helps me prove I’m not just lazy and crazy. I just hurt. A lot.”

“I think the education this program provides will empower so many people who have no resources or support and suffer from this on a day-to-day basis. I think the intention and purpose behind all of the curriculum is incredibly inspiring and such a hopeful solution for those of us who need it!”

“Would probably have rated it higher when I was first injured. At 12+ years, I’ve seen it all before…”

“Shockingly accurate.”

“These are very clearly written and visually explained.”

“It is how I feel.”

“Your grasp of the syndrome is understood, well thought out and explained.”

“Such great information!”

#### 3.5.3 Suggested edits to the *SeePain*

Participants also provided suggested edits to the *SeePain*. Overall, their comments highlighted some important edits that could be made to improve the content of this educational tool. Participants stated that it would be helpful to include some discussion regarding future pain management strategies and the use of cannabis. They also suggested that diagrams could be more detailed, as well as detailed explanations according to the level of injury and the experience of pain.

“Some discussion of the future of pain management might give hope to patients.”

“How to get off pain meds that have side effects and recommendations to alleviate pain are the most helpful. Do not need explanations of what pain is!”

“… Any info or studies on any aspects of THC pain management would be super helpful and I would love to participate in any way I could.”

“Wished it was more specific and went into more detail. Also, include each level of injury and what is affected.”

“Diagrams could use more detail.”

“Terminology explanations could be described in Layman’s terms.”

“… Seems far too positive about opioids. Could be more positive about cannabis given that it seems to have little in the way of bad side effects (unlike the other meds discussed…)”

We would like to indicate that the themes that emerged in the comments from both the present and previous publications (9) are similar. However, participants who took part in the current survey outlined in this manuscript received a newly revised version of the SeePain based on previous participants’ comments from our 2023 publication.

### 3.6 Exploratory factor analysis for perceived agreement and usefulness statements

An exploratory factor analysis was performed, including the perceived agreement and usefulness scores for the *SeePain* evaluative statements. The analysis resulted in four factors/components that accounted for 67.23% of the total variation. These factors were labeled as (1) Content, (2) Clarity, (3) Delivery medium, (4) Format. The KMO measure of sampling adequacy confirmed the adequacy of perceived agreement and usefulness ratings for the factor analysis (0.877) together with the significant Bartlett’s test of sphericity (*χ*^2^ = 1621.852; *p* < 0.001). The factor labeled “clarity” was found to have the largest factor loadings, indicating that most completers considered it to be a crucial aspect within the perceived agreement and usefulness dimensions (see Table 5).

**Table 5:** Perceived agreement and usefulness dimensions (*N* = 138).

Pattern matrix^*^				
Perceived agreement and usefulness themes	Factors/Components			
	Content	Clarity	Delivery medium	Format
The explanations regarding pain and how it happens are clear.		0.94^†^		
The pain types that are common and how they develop over time after SCI are well explained.		0.90^†^		
The language is understandable throughout the *SeePain*.		0.94^†^		
Figures and tables are clear.		0.91^†^		
Self-management of pain	0.79^†^	0.13		0.14
Factors that make pain worse	0.77^†^			
Cannabis/opioids	0.59^†^		0.18	
Role of psychological factors	0.92^†^			
Pain medication	0.62^†^	0.18	0.18	
SCI-related pain	0.83^†^			0.12
Support for significant other/family	0.59^†^		0.13	0.17
Electronic (current version)		0.11		0.78^†^
Website version				0.78^†^
Short videos			0.68^†^	0.38
In-person learning			0.84^†^	
App-based learning		0.10	0.79^†^	0.11
How useful would it be to have a shorter version of the *SeePain*?				0.69^†^
How useful would the *SeePain* be to help you discuss your pain with your significant other and family members?	0.64^†^		0.32	
How useful would the *SeePain* be to help you to discuss your pain with your doctor and other healthcare professionals?	0.64^†^		0.28	

^*^Extraction method: principal component analysis; rotation method: promax with Kaiser normalization (factor loadings <0.10 not shown). ^†^Primary loadings (paper version, format of the seepain is good, overall content is useful and relevant, and healthcare provider perspectives themes excluded, cross-loading < 0.15 present).

### 3.7 Group comparisons on perceived agreement and usefulness factor scores

The analyses showed that individuals with higher education (college graduates *N* = 82, Mean = 0.14; SD = 1) had significantly (*t* (136) = −2.122, *p* = 0.036, Cohen’s d = −0.36) greater factor scores on the *format* factor compared to no college graduates (*N* = 56, Mean = −0.2; SD = 0.9), suggesting that they were more likely to endorse electronic and website formats, and usefulness of a shorter version of the *SeePain* to a greater degree than non-college graduates. For gender and age, we found that females (*N* = 51, Mean = 0.26; SD = 0.7) and younger individuals (*N* = 45, Mean = 0.22; SD = 0.69) demonstrated significantly higher factor loadings on the *clarity* factor compared with males (*N* = 85, Mean = −0.15; SD = 1) (*t* (134) = 2.354, *p* = 0.020, Cohen’s d = 0.41), and older individuals ≥ 46 years old (*N* = 93, Mean = − 0.10; SD = 1) (*t* (136) = 2.149, *p* = 0.034, Cohen’s d = 0.33). These results indicate greater endorsement of clarity for comprehension and understanding of pain explanations, its progression over time, language, figures, and tables, in females and younger individuals. For the level of injury (cervical and below-cervical), we did not find significant differences between cervical and below-cervical groups in each of the factor scores: *content* (*t* = 1.231, *p* = 0.11); *clarity* (*t* = 0.534, *p* = 0.06). Similarly, non-significance was found for the presence of neuropathic pain [0–1 SCIPI (no neuropathic pain) and 2–4 SCIPI (no neuropathic pain)]: *content* (*t* = −0.738, *p* = 0.21); *clarity* (*t* = 0.206, *p* = 0.95).

Finally, one-way ANOVAs revealed no significant differences between years living with pain and the factors scores (*clarity*: *F* (2, 135) = 2.45, *p* = 0.09; *content*: *F* (2, 135) = 2.54, *p* = 0.08). Thus, information regarding pain appears to be perceived as equally useful at any time range of living with pain. Comparably, another analysis with one-way ANOVAs revealed no significant differences between years living with SCI and the factors scores (*clarity*: *F* (2, 135) = 1.916, *p* = 0.15; *content*: *F* (2, 135) = 1.327, *p* = 0.27), as well as ethnicity and the factor components (*clarity*: *F* (2, 135) = 0.845, *p* = 0.43; *content*: *F* (2, 135) = 1.801, *p* = 0.17).

### 3.8 Correlations

A significant positive correlation was observed between *content* factor and pain intensity (*r* = 0.2, *p* = 0.041), suggesting that individuals with more intense pain had higher scores on the content of *SeePain*. However, no significant correlations were detected with the pain severity subscale, pain interference, and difficulty living with pain.

## 4 Discussion

The current survey study evaluated the agreement and usefulness ratings of statements regarding clarity/comprehensibility, content, and format of a two-module educational resource for people with SCI, the *SeePain*. The primary purpose was to determine the overall utility, underlying dimensions, and any associations with pain or demographic characteristics in a larger sample of people with SCI-related non-neuropathic pain (*N* = 138). The responses indicated high levels of agreement with the comprehensibility and clarity of this resource, with the language being reported as highly understandable, figures and tables as clear, and content perceived as useful and relevant. Furthermore, the perceived usefulness of the *SeePain* was moderate to high, with the highest level of usefulness reported for the modules discussing SCI-related pain and self-management of pain. These results indicate that the content of the *SeePain* was well understood by our respondents and provided specific and clear information related to their condition.

When examining the pain characteristics, we found that 77% of those who completed all the survey questions were classified as experiencing neuropathic pain according to the SCIPI cutoff criteria of two or higher. This is consistent with the prevalence of neuropathic pain of 60–70% in the SCI population (1). Completers reported that their pain intensity was moderately high, emphasizing the significant impact of pain and its distressing nature among those included in the study. They also consistently reported a moderate level of difficulty based on pain severity, total pain interference, and challenges in dealing with pain.

A subsequent factor analysis of the agreement and perceived usefulness ratings to determine underlying dimensions resulted in four factors/components: (1) Content, (2) Clarity, (3) Delivery medium, and (4) Format. Results from our factor analysis suggested that most of the variation associated with the *SeePain’s* could be attributed to *content*. The areas with factor loadings that best represented *content* (>0.7) were related to psychological factors, SCI-related pain, self-management of pain, and factors that make pain worse. Additionally, the factor *clarity* was highly represented by factor scores (>0.90) related to understandable language, clarity of explanations, figures and tables, and information about the emergence of pain and how it progresses over time.

Interestingly, educational background, gender, and age were shown to be significantly influential in participants’ perceptions of the *SeePain* as a resource. For example, individuals with higher education had greater factor scores on electronic and website formats, and usefulness of a shorter version of the *SeePain* than non-college graduates. Females and younger individuals had greater factor scores for clarity of the *SeePain,* that is, comprehension and understanding of pain explanations, its progression over time, language, figures, and tables. Additionally, a significant correlation was found between pain intensity and content; that is, individuals with more intense pain had higher factor scores on the *content* factor of the *SeePain*, suggesting that they perceived the content to be most useful. However, neither years of living with SCI and pain nor pain severity had any impact on the different components of this educational material, suggesting that information regarding pain is perceived equally useful at any time range of living with pain and its severity. Our results align with previous research studies, which show that individuals with persistent pain benefit from learning more about their condition through their participation in a structured program (15–17). However, we also found that format and clarity were associated with greater factor scores for those with higher education and younger individuals. This may suggest that educational material regarding pain after SCI, both content and format, may need to be tailored to age and level of education.

Studies incorporating pain education as part of a comprehensive pain management program have been shown to help mitigate ratings of pain intensity and pain-related disability (8), mood (6), and select measures of life control in those living with SCI associated neuropathic pain (7). Those with chronic pain conditions who demonstrate lower measures of health literacy also tend to report less knowledge about pain medication and where to find adequate care for their pain symptoms (18, 19). The latter point is particularly concerning given that those with SCI are more likely to be long-term opioid users (20). This may produce limited pain relief and elevate the risk for additional health complications (21) — both of which may be mitigated through education about adverse drug effects. In addition, lower health literacy has been associated with poorer disease-related knowledge and beliefs about pain and lower non-emergency medical use (22). Indeed, individuals with SCI often report dissatisfaction with the knowledge and information given by their healthcare providers on SCI-related pain (4). Therefore, our findings suggest that the *SeePain* represents a concise educational resource about the intricacies of pain and potential treatment options for those with SCI while adding individual consumer perspectives regarding the lived experience. Furthermore, it may also serve as an introductory overview of self-management pain treatment strategies that may be beneficial both for those with SCI and their caregivers.

Additionally, the *SeePain* has been structured based on scientific literature and stakeholders’ input, providing pain educational content for individuals with SCI, their significant others, and healthcare providers. Therefore, given its multidimensional structure, the positive comments, and the reported ease at which the information was interpreted highlight the value of this resource within this heterogeneous and often disadvantaged clinical population. This is consistent with evidence showing that the management of neuropathic pain can be difficult and may require a multidimensional approach (23), as well as “interdisciplinary pain programs involving patient education, cognitive behavioral therapy, self-management strategies, group discussions, exercise, and other modalities” (24).

Finally, the comments provided by participants also highlighted their perspectives on the included content and potential usefulness of the *SeePain*, including some suggestions to improve its overall format. That said, some survey completers emphasized that the *SeePain* contained information that they could not previously put into words. Some also reported that such information would help empower those with pain to participate in their care and make more informed medical decisions. Some asked for more information about cannabis and effective ways to manage side effects associated with commonly prescribed pain medications. Given the increasing interest in the use of cannabis and/or cannabinoids for neuropathic pain (25, 26), future research should continue to examine the benefits and potential harms associated with such substances within this population.

### 4.1 Limitations

It is important to note potential limitations to the current study that may affect the generalizability of the current findings. *First*, the design of the survey did not allow participants to report a specific age but asked them to select one of four age ranges that applied to them. As a result, the majority reported being between 46 and 60 years old, and consequently, limited our ability to optimally explore potential associations between factor scores and age as a continuous variable. *Second,* most participants were white/caucasian. Thus, there was an under-representation of other ethnicities. *Third*, most participants had a college or an advanced degree. This may not adequately represent the general SCI population. Therefore, future research should explore specific educational approaches and formats for those with lower educational levels. *Finally*, there may have been a selection bias as the survey required prolonged access to the internet. Subsequently, people from a lower socioeconomic background may not have had ready access to the internet and thus faced an additional barrier to entering or completing the study.

## 5 Conclusion

In summary, the present study demonstrated that people with SCI who experienced chronic pain found the *SeePain* to be useful. Therefore, the *SeePain* may be a valuable resource across clinical settings, research settings, and community groups. The content could be delivered in shorter sections targeting specific topics and shared via social media through podcasts, video segments, or infographics to facilitate a greater reach of the material to all of those who may find it beneficial. We are currently developing a Spanish version of the *SeePain* for wider distribution, and are utilizing the current version in our research studies that include educational group sessions focused on neuropathic pain. Finally, our results may inform the development of similar educational material tailored to specific populations who may not have access to quality pain information and ways to better manage their symptoms.

## Data availability statement

The raw data supporting the conclusions of this article will be made available by the authors, without undue reservation.

## Ethics statement

The studies involving humans were approved by Institutional Review Board of the University of Miami Miller School of Medicine. The studies were conducted in accordance with the local legislation and institutional requirements. The first part of the survey consisted of an information page and an institutionally approved waiver of signed informed consent. Consent was granted by the agreement to continue with the survey.

## Author contributions

GF: Conceptualization, Data curation, Formal analysis, Investigation, Methodology, Writing – original draft, Writing – review & editing. KA: Conceptualization, Data curation, Funding acquisition, Investigation, Project administration, Resources, Supervision, Writing – original draft, Writing – review & editing. RV: Formal analysis, Methodology, Writing – original draft, Writing – review & editing. SF: Formal analysis, Methodology, Writing – original draft, Writing – review & editing. LR: Formal analysis, Methodology, Writing – original draft, Writing – review & editing. NC: Formal analysis, Methodology, Writing – original draft, Writing – review & editing. WK: Methodology, Supervision, Writing – review & editing. EW-N: Conceptualization, Funding acquisition, Investigation, Project administration, Resources, Supervision, Writing – original draft, Writing – review & editing.

## Footnotes

^1^https://www.surveymonkey.com/mp/legal/security/
