# Supplementary material for: Perspectives of people with spinal cord injury on a pain education resource
Source: Front Public Health. 2024 Jun 19;12:1385831. doi: 10.3389/fpubh.2024.1385831 (PMC11220275; doi:10.3389/fpubh.2024.1385831)
Supplement: Supplementary file 1 [file Data_Sheet_1.PDF]

SeePain Survey

Information and Consent

Development of pain education for improving pain health literacy and quality of life after spinal cord injury

The primary purpose of this research study is to refine a newly developed educational resource (the SeePain) regarding the pain issues that people often experience following their spinal cord injury (SCI). Our previous work was based on consumer input and showed that many consumers do not feel that they received adequate information from their healthcare providers regarding pain and that most viewed provider-patient communication and educational efforts regarding pain and its management as critical needs.

In order to finalize the SeePain and make it optimally useful we need to better understand SCI consumer perspectives on the content, relevance, comprehensiveness, and format of the SeePain. The goal of this proposal is to develop a relevant, consumer-grounded educational resource that will be made available to the general SCI community, their families, and their health care providers.

To participate in this study you must have an SCI and have experienced moderate to severe SCI-related pain for six months or longer. You must be between 18-70 years of age and be able to understand written English language.

You will be asked to complete an online questionnaire that asks about several aspects of living and coping with SCI and persistent pain. The questionnaire asks for some basic information about you, such as age and gender, and the details of your SCI and your day-to-day pain experiences. The questionnaire will also ask you about your perspectives on the SeePain, which will be available to you. The time to complete the questionnaire is 20 - 30 minutes.

There are no benefits or monetary compensation for participating in this survey. However, by participating, you may obtain access to the SeePain educational resource.

Participation is completely voluntary. As such, you may decline to participate or you can stop participating at any time without any negative consequences to you. However, if you do not complete the questionnaire, your answers will not be saved for use in this research study.

The research information you share with us in the course of completing the questionnaire will not contain individually identifiable private information and this information will only be identified by way of a code that is not based on information that identifies you. The Principal Investigator, her collaborators and staff, the Sponsor (Craig Neilsen Foundation), and SurveyMonkey™ (the Web-based application being used to collect your responses) will consider your information confidential to the extent permitted by law. Click here to review the security

measures in place at SurveyMonkey™.

If at any time you have any questions about the study, please contact the study doctor, Dr. Eva Widerström-Noga at (305) 243-7125.

Your decision to complete the questionnaire is your consent to participate in this research.

* 1. Do you choose to continue this survey?

◯ Yes

◯ No

SeePain Survey

Questions About Your Pain

After SCI you can have several different types of pain in different areas of your body. We ask you to think about your OVERALL PAIN when answering the questions below.

* 2. Please rate your average pain intensity of ALL your pain problems in the past week

0 - no pain	1	2	3	4	5	6	7	8	9	10 - pain as bad as you can imagine
◯	◯	◯	◯	◯	◯	◯	◯	◯	◯	◯

* 3. Overall, how hard is it for you to deal with your pain?

0 - not hard at all	1	2	3	4	5	6	7	8	9	10 - extremely hard
◯	◯	◯	◯	◯	◯	◯	◯	◯	◯	◯

* 4. Rate the level of your pain at the present moment.

0 - no pain	1	2	3	4	5	6 - very intense pain
◯	◯	◯	◯	◯	◯	◯

* 5. On the average, how severe has your pain been during the past week?

0 - not at all severe	1	2	3	4	5	6 - extremely severe
◯	◯	◯	◯	◯	◯	◯

* 6. How much suffering do you experience because of your pain?

0 - no suffering	1	2	3	4	5	6 - extreme suffering
○	○	○	○	○	○	○

* 7. In general, how much has pain interfered with your day-to-day activities in the last week?

0 - no interference	1	2	3	4	5	6	7	8	9	10 - extreme interference
○	○	○	○	○	○	○	○	○	○	○

* 8. In general, how much has pain interfered with your overall mood in the last week?

0 - no interference	1	2	3	4	5	6	7	8	9	10 - extreme interference
○	○	○	○	○	○	○	○	○	○	○

* 9. In general, how much has pain interfered with your ability to get a good night's sleep in the last week?

0 - no interference	1	2	3	4	5	6	7	8	9	10 - extreme interference
○	○	○	○	○	○	○	○	○	○	○

* 10. Is the quality of pain electrical or electric shock like?

○ Yes

○ No

* 11. Is the quality of pain like pins and needles, or tingling?

○ Yes

○ No

* 12. Does the skin over the area of pain or inside your body where the pain is located feel hot or burning or cold or freezing?

○ Yes

○ No

* 13. Does the pain only occur in an area of the body in which you have no feeling on the skin overlying that area?

○ Yes

○ No

SeePain Survey

Questions About You and Your Injury

We would like to know about you and your type of injury. Please answer the following questions.

* 14. How old are you? (years)

- ○ 18-30
- ○ 31-45
- ○ 46-60
- ○ 61 or older

* 15. How many years have you lived with a spinal cord injury?

[dropdown]

* 16. What is the highest level of education you have completed?

[dropdown]

* 17. How many years have you lived with pain?

[dropdown]

* 18. What is your gender? (not your sexual orientation)

- ○ Female
- ○ Male
- ○ Non-binary
- ○ Transgender

* 19. What is your ethnicity? (Please choose all that apply.)

- ☐ American Indian or Alaskan Native
- ☐ Asian
- ☐ Black or African American
- ☐ Hispanic or Latino
- ☐ Native Hawaiian or Other Pacific Islander
- ☐ White / Caucasian

Other (please specify)

[text box]

* 20. Where is the main area that your spinal cord injured?

- ◯ Neck
- ◯ Below the neck

* 21. Do you have any feeling or voluntary movement below your level of injury?

- ◯ Yes, I have some voluntary movement
- ◯ Yes, I have some feeling
- ◯ Yes, I have some feeling and voluntary movement
- ◯ I have no feeling or voluntary movement below my level of injury

SeePain Survey

Questions About SeePain

There are 2 chapters of SeePain. Click on each link below to open the SeePain chapters in another window.

Chapter 1
Chapter 2

After reading through the SeePain, please rate the statements on the following pages.

SeePain Survey

Comprehensibility/Clarity

* 22. The explanations regarding pain and how it happens are clear.

0 - completely disagree	1	2	3	4	5	6	7	8	9	10 - completely agree
◯	◯	◯	◯	◯	◯	◯	◯	◯	◯	◯

* 23. The pain types that are common and how they develop over time after SCI are well explained.

0 - completely disagree	1	2	3	4	5	6	7	8	9	10 - completely agree
◯	◯	◯	◯	◯	◯	◯	◯	◯	◯	◯

* 24. The language is understandable throughout the SeePain.

0 - completely disagree	1	2	3	4	5	6	7	8	9	10 - completely agree
○	○	○	○	○	○	○	○	○	○	○

* 25. Figures and tables are clear.

0 - completely disagree	1	2	3	4	5	6	7	8	9	10 - completely agree
○	○	○	○	○	○	○	○	○	○	○

26. Comments

SeePain Survey

Content

* 27. The overall content is useful and relevant.

0 - completely disagree	1	2	3	4	5	6	7	8	9	10 - completely agree
○	○	○	○	○	○	○	○	○	○	○

* 28. Please indicate the areas that were most useful and least useful to you.

	0 - least useful	1	2	3	4	5	6	7	8	9	10 - most useful
Healthcare provider perspectives	○	○	○	○	○	○	○	○	○	○	○
Self-management of pain	○	○	○	○	○	○	○	○	○	○	○
Factors that make pain worse	○	○	○	○	○	○	○	○	○	○	○
Cannabis/opioids	○	○	○	○	○	○	○	○	○	○	○
Role of psychological factors	○	○	○	○	○	○	○	○	○	○	○
Pain medication	○	○	○	○	○	○	○	○	○	○	○
SCI-related pain	○	○	○	○	○	○	○	○	○	○	○
Support for significant other/family	○	○	○	○	○	○	○	○	○	○	○

29. Comments

SeePain Survey

Format

* 30. The format of the SeePain is good.

0 - completely disagree	1	2	3	4	5	6	7	8	9	10 - completely agree
○	○	○	○	○	○	○	○	○	○	○

* 31. Please indicate the areas that would be most useful and least useful to you.

	0 - least useful	1	2	3	4	5	6	7	8	9	10 - most useful
Paper version	○	○	○	○	○	○	○	○	○	○	○
Electronic (current version)	○	○	○	○	○	○	○	○	○	○	○
Website version	○	○	○	○	○	○	○	○	○	○	○
Short videos	○	○	○	○	○	○	○	○	○	○	○
In-person learning	○	○	○	○	○	○	○	○	○	○	○
App-based learning	○	○	○	○	○	○	○	○	○	○	○

* 32. How useful would it be to have a shorter version of the SeePain?

0 - least useful	1	2	3	4	5	6	7	8	9	10 - most useful
○	○	○	○	○	○	○	○	○	○	○

* 33. How useful would the SeePain be to help you discuss your pain with your significant other and family members?

0 - least useful	1	2	3	4	5	6	7	8	9	10 - most useful
○	○	○	○	○	○	○	○	○	○	○

* 34. How useful would the SeePain be to help you to discuss your pain with your doctor and other healthcare professionals?

0 - least useful	1	2	3	4	5	6	7	8	9	10 - most useful
○	○	○	○	○	○	○	○	○	○	○

SeePain Survey

Thank you! You have completed this survey.

For information about more Miami Project research please visit **www.themiamiproject.org/participant/research-participation/clinical-trials-and-research-studies/**
